# Association du polymorphisme de la méthylènetétrahydrofolate réductase C677T avec le risque de cancer colorectal sporadique

**DOI:** 10.11604/pamj.2021.38.287.12522

**Published:** 2021-03-18

**Authors:** Imane Baghad, Driss Erreguibi, Rachid Boufettal, Saad Rifki Eljai, Farid Chihab, Sellama Nadifi

**Affiliations:** 1 Laboratoire de Génétique et Pathologie Moléculaire, Faculté de Médecine et Pharmacie, Université Hassan II de Casablanca, Casablanca, Maroc,; 2 Service de Chirurgie Viscérale Aile III, Centre Hospitalier Universitaire Ibn Rochd, Université Hassan II, Casablanca, Maroc

**Keywords:** Gène MTHFR, polymorphisme C677T, cancer colorectal, sporadique, Maroc, population, MTHFR gene, C677T polymorphism, colorectal cancer, sporadic, Morocco, population

## Abstract

Le cancer colorectal (CCR) est un problème majeur de santé publique dans le monde. Le métabolisme des folates est impliqué dans la synthèse, la réparation et la méthylation de de l´ADN. La méthylènetétrahydrofolate réductase (MTHFR) est une enzyme clé dans le métabolisme folique. Un polymorphisme commun de la MTHFR, le C677T, a été corrélé aux CCR. Cette étude cas témoins a été menée pour analyser l´association entre ce polymorphisme et le risque de CCR sporadique dans une population marocaine. Soixante-seize (76) patients atteints de cancer colorectal sporadique confirmé histologiquement et 182 témoins sans antécédents de cancer ont été recrutés pour cette étude. L´ADN a été isolé à partir de sang périphériques et les génotypes ont été déterminés par PCR-RFLP. Le risque d´association a été estimé par le calcul de l´odds ratio (OR) avec un intervalle de confiance à 95%. La fréquence des génotypes MTHFR chez les patients et les témoins a été de 34.1%CC; 56.6%CT; 9.21%TT et 51,6%CC; 42,8% CT; 6% TT respectivement. Le génotype CT et sa combinaison avec le génotype TT et l´allèle T sont associés à un risque accru de CRC avec un OR de 2.02 (avec un intervalle de confiance (IC) de 95%: 1.14-3.58, p = 0,01), de 2.05 (IC 95%: 1.18-3.58, p = 0.01) et de 1.61 (IC 95%: 1,07-2.40, p = 0,02). Les homozygotes TT n´ont pas été un facteur de protection dans notre étude avec un OR de 2,30 (IC 95%: 0.81-6.52, p = 0,11). Il existe une association statistiquement significative entre le variant MTHFR C677T et le risque de survenue de cancer colorectal sporadique dans la population étudiée.

## Abstract (en)

Colorectal cancer (CRC) is a major global public health problem. Folate metabolism is involved in DNA synthesis, repair and methylation. Methylenetetrahydrofolate reductase (MTHFR) is a key enzyme in folate metabolism. Common MTHFR C677T polymorphism was correlated to CRC. This case-control study was conducted to analyze the association between this polymorphism and the risk of sporadic CRC in a Moroccan population. The study involved 76 patients with sporadic colorectal cancer confirmed histologically and 182 patients (control group) without a history of cancer. Deoxyribonucleic acid (DNA) was isolated from peripheral blood and genotypes were determined using PCR-RFLP. The risk of association was estimated using odds ratio (OR) with 95% confidence interval. Genotype frequency of MTHFR in patients and in the control group was CC 34.1%, CT 56.6%, TT 9.21%, CC 51.6%, CT 42.8% and TT 6% respectively. CT genotype and its combination with TT genotype and allele T were associated with an increased risk of CRC and with an OR of 2.02 (with 95% confidence interval [CI]: 1.14-3.58, p = 0.01), 2.05 (95 % CI: 1.18-3.58, p= 0.01) and 1.61 (95% CI: 1.07-2.40, p=0.02). Homozygous TT weren´t a protection factor in our study, with an OR of 2.30 (95% CI: 0.81-6.52, p = 0.11). There was a statistically significant association between the MTHFR C677T variant and the risk of occurrence of sporadic colorectal cancer in the studied population.

## Introduction

Le cancer colorectal (CCR) représente un enjeu majeur de santé publique dans le monde. Il représente le troisième cancer le plus fréquent au Maroc. Son taux d´incidence standardisée est estimé à 11,9 pour 100000 habitants dans la population marocaine contre 14,7 pour 100000 habitants dans la population mondiale avec une légère prédominance masculine [1]. Le CCR est sujet à de nombreuses études génétiques au cours de ces dernières années, le but étant de trouver des tests génétiques assez puissant pour permettre un diagnostic précoce voir un dépistage primaire de cette maladie, ainsi qu´une meilleure compréhension et utilisation des moyens thérapeutiques. Les CCR sporadiques constituent un groupe d´affection maligne hétérogène car ils sont déterminés à la fois par des facteurs génétiques et environnementaux [2]. Plusieurs études ont porté sur le rôle des folates dans la genèse du CCR en raison de leur rôle prédominant dans la prolifération cellulaire, en effet ils interviennent dans la synthèse, la réparation de l´ADN et la régulation de l´expression des gènes spécifiques en particulier par des phénomènes épigénétiques (méthylation de l´ADN) [3]. Une des enzymes clé du métabolisme des folates dans l'organisme est la méthylènetétrahydrofolate réductase (MTHFR). Cette enzyme catalyse la réduction irréversible du 5,10-méthylènetétrahydrofolate (5,10-méthylène-THF) en 5-méthyl-THF qui sert de substrat pour la reméthylation de l'homocystéine en méthionine source de radicaux méthyl chez l´être humain [4].

La 5-méthyl-THF joue aussi un rôle dans la synthèse des pyrimidines (ADN). En outre, le cycle des folates contribue à la synthèse de purine (ADN) et d'acide ribonucléique (ARN) [5]. Le polymorphisme du gène MTHFR le plus souvent étudié est le C677T, il est impliqué dans plusieurs troubles multifactoriels incluant la pathologie néoplasique. Chez le sujet homozygote, l´activité enzymatique de la MTHFR est diminué de 70% et de 35% chez l´hétérozygote CT, cette déficience entraine une hyperhomocystéinémie responsable d´une hypométhylation global de l´ADN ce qui pourrait favoriser le développement du CCR [6]. Il a été démontré qu´un apport alimentaire faible en folate et en méthionine associé à un alcoolisme chronique peut augmenter le risque de CCR [7,8]. Ce risque est encore plus grand lorsque le sujet à un génotype TT du gène MTHFR [9]. Cependant, plusieurs études ont démontré que le génotype 677TT associé à un apport suffisant en folates aurait un effet protecteur et serait associé à un risque moindre de CCR [10,11]. Alors que d'autres études n'ont montré aucune association [12,13]. Par conséquent, les résultats obtenues jusqu´à aujourd'hui sont variables d´une étude à l´autre, ils n´ont pas tranché clairement en faveur de l´effet protecteur ou délétère du polymorphisme C677T de la MTHFR sur la survenue du CCR. Il nous a paru donc évident de mener une étude de type cas-témoins dont l´objectif principal est d´étudier l´association du polymorphisme C677T de la MTHFR et le risque de CCR sporadique dans une population marocaine.

## Méthodes

C´est une étude cas-témoin colligée au service de chirurgie viscérale Aile III du Centre hospitalier Ibn Rochd de Casablanca entre juin 2013 et novembre 2015. Il s´agissait de 76 patients atteints d'un cancer colorectal sporadique à différents stades d'évolution, le diagnostic était confirmé par un examen anatomopathologique, un consentement éclairé était signé par tous les patients et les témoins. L'étude pratique a été effectuée au niveau du service de génétique et pathologie moléculaire de la faculté de médecine de Casablanca. Le groupe de témoin était composé de 182 sujets issus de la population générale ne présentant aucune pathologie maligne. L‘extraction de l´ADN génomique par la technique aux sels standard était réalisé à partir d´un prélèvement sanguin de 5 ml recueilli dans un tube EDTA. La qualité et la quantité de L'ADN extrait était contrôlé sur un gel d'agarose à 1% en utilisant un spectrophotomètre. Les génotypes du polymorphisme du gène MTHFR (C677T) était détectés par une réaction de polymérase en chaine (PCR) suivi d´une digestion enzymatique par une enzyme de restriction HINF1 (RFLP). L'analyse statistique a été effectuée à l'aide du programme statistique SPSS 11.0 (SPSS Inc., Chicago, IL, USA) et le logiciel convivial Med-Calc 11.5.1.0. Le test de l´équilibre Hardy-Weinberg pour le polymorphisme C677T MTHFR a été effectué séparément dans les cas et les contrôles. L´analyse de la relation entre les caractéristiques cliniques et histologiques et les différents génotypes a été réalisée par le test de chi-carré (χ^2^). Une valeur P inférieure à 0,05 était considéré comme significative. Les associations entre les génotypes et le risque de CRC ont été estimés en calculant l´Odds ratio (OR) avec des intervalles de confiance (IC) de 95%. Le risque de développer un cancer a été calculé à partir du Risque Relatif (RR).

## Résultats

L'étude statistique a montré que les deux groupes (cas et témoins) se trouve dans l´équilibre de Hardy Weinberg pour la distribution du polymorphisme MTHFR (X^2^= 0.80, P = 0,37; X^2^= 3.2, P = 0.10). La corrélation entre le polymorphisme MTHFR et les caractéristiques clinico-pathologiques, a montré aucune différence significative entre les deux groupes par rapport à l'âge, au sexe, aux antécédents familiaux, à la localisation et au type histologique de la tumeur (Tableau 1). La fréquence des génotypes trouvées chez les patients et les témoins étaient de 34.1%CC; 56.6%CT; 9.21%TT et 51,6% CC, 42,8% CT, 6% TT, respectivement. Les fréquences alléliques des allèles C et T étaient de 62.5% et 37.5% pour les patients et 72.8% et 28.2% pour les témoins. Nos résultats montrent que l'allèle T et l'hétérozygote CT sont associé significativement à un risque plus élevé de CCR avec un OR de 1.61 (IC 95%: 1,07-2.40, p = 0,02) et 2.02 (IC de 95 %: 1.14-3.58, p = 0,01), respectivement. La combinaison des génotypes CT et TT était statistiquement significatif avec un OR de de 2.05 (IC 95 %: 1.18-3.58, p= 0.01). Nos résultats ont démontré que l´homozygote TT n'était pas un facteur protecteur du CCR avec un OR de 2,30 (IC 95%: 0.81-6.52, p = 0,11) et que le polymorphisme MTHFR C677T peut augmenter le risque de CCR dans la population marocaine (Tableau 2). Le risque Relatif d'avoir la maladie était significativement plus élevé chez les individus porteurs d'au moins un allèle T (TT ou CT) que les porteurs CC avec un RR de 1,38 (IC 95% : 1.06-1.80, p =0,01) (Tableau 3).

**Tableau 1:** fréquence génotypique du polymorphisme C677T MTHFR chez les patients atteints de CCR sporadique selon les caractéristiques clinico-pathologiques

	N	C677T MTHFR				Chi-square Test
		CC (%)	CT (%)	TT (%)	P value	
**Age**				5(14.7) 2 (4.8)	0.28	2.51
<45	34	12(35.3)	17(50.0)			
≥45	42	14 (33.3)	26 (61.9)			
**Sexe**					0.4	1.81
homme	40	14(35.0)	24 (60.0)	2(5.0)		
femme	36	12(33.3)	19(52.8)	5(13.9)		
**Délai diagnostic**					0.42	1.76
>6 mois	42	12 (28.6)	25 (59.5)	5 (11.9)		
≤6 mois	34	14 (41.2)	18 (52.9)	2 (5.9)		
**Antécédent Familial**					0.44	3.79
CCR	12	2 (16.7)	8 (66.7	2 (16.7)		
Autre cancer	5	1 (20.0)	3 (60.0)	1 (20.0)		
Pas ATCD	59	23 (39.0)	32 (54.2)	7(9.2)		
**Localisation**					0.38	8.57
Colon droit	14	5 (35.7)	8 (57.1)	1 (7.1)		
Colon Transverse	2	1 (50.0)	1 (50.0)	0 (0)		
Colon gauche	16	9 (56.2)	7 (43.8)	0 (0.0)		
Sigmoïde	28	7 (25.0)	16 (57.1)	5 (17.9)		
Rectum	16	4 (25.0)	11 (68.8)	1 (6.2)		
**Composante histologique**					0.73	0.62
ADK simple	59	19 (32.2)	34(57.6)	5 (10.2)		
ADK mucineux	17	7 (41.2)	9 (52.9)	7 (9.2)		
**Différenciation**					0.22	5.71
Bien	27	5 (18.5)	19 (70.4)	3 (11.1)		
Modéré	36	16 (44.4)	18 (50.0)	2 (5.6)		
Peu	13	5 (38.5)	43 (56.6)	7 (9.2)		
**Stade**					0.28	7.42
Stade I	11	2 (18.2)	9 (81.8)	0 (0)		
Stade II	21	6 (28.6)	12 (57.1)	3 (14.3)		
Stade II	28	9 (32.1)	16 (57.1)	3 (10.7)		
Stade IV	16	9 (56.2)	6 (37.5)	1 (6.2)		

CCR: cancer colorectal, N: nombre; MTHFR génotype: homozygote (CC); hétérozygote (CC); mutant homozygote (TT)

**Tableau 2:** fréquences génotypiques et alléliques du polymorphisme C677T du gène MTHFR chez les patients atteints de cancer colorectale sporadique

MTHFR (C677T) génotype	Cas (%) N 76	Témoin (%) N 182	OR (95% CI)	P
CC	26(34.1%)	94(51.6%)	Réf	
CT	43 (56.6%)	77(42.8%)	2.02 (1.14-3.58)*	0,01*
TT	7 (9.21%)	11(6.0%)	2.30 (0.81-6.52)	0,11
CT+TT	50 (65.8%)	88(48.3%)	2.05 (1.18-3.58)*	0,01*
C	95 (62.5%)	265(72.8%)	Réf	
T	57 (37.5%)	99(27.2%)	1,61(1.07-2.40)*	0,02*

CC: MTHFR C677T variant homozygote, CT: MTHFR C677T variant hétérozygote, TT: MTHFR C677T variant homozygote, N: nombre total, OR: odd ratio, P: p value (*P<0.05), Ref: reference

**Tableau 3:** risque relatif des cas comparé aux témoins

MTHFR (C677T) génotype	Cas (%) N 76	Contrôles (%) N 182	RR (95% CI)	P
CC	26 (34.1 %)	94(51.6%)	Ref	
CT	43 (56.6%)	77(42.8%)	1.38 (1.08-1.77)*	0,01*
TT	7 (9.21%)	11(6.0%)	2,02 (0.85-4.80)	0,10
CT+TT	50 (65.8%)	88(48.3%)	1.36 (1.09 -1.69)*	0,006*
C	95 (62.5%)	265(72.8%)	Ref	
T	57 (37.5%)	99(27.2%)	1,38(1.06-1.80)*	0,01*

RR: risque relative * P<0.05

## Discussion

Le CCR est le premier cancer du tube digestif au Maroc [1]. L´étiopathogénie du CCR sporadique est toujours mal comprise. Cependant plusieurs études ont démontré l´association de gènes prédisposant à un risque élevé ou diminué de survenue de CCR. La première étude évaluant l´association du polymorphisme C677T de la MTHFR et le risque de CCR a été menée par Chen *et al*. [14]. En effet, elle suggère que la mutation C677T est impliquée dans la synthèse, la réparation et la méthylation de l´ADN induisant une tumorogénèse colorectale. Cependant, Les résultats obtenus par différentes équipes restent contradictoires [15]. Nous avons mené cette étude pour évaluer l'interaction entre le gène MTHFR C677T et le CCR sporadique dans la population marocaine. En comparant les caractéristiques clinico-pathologiques entre les patients de différents génotypes, aucune différence significative n´a été observée par rapport à l´âge, le sexe, le délai de diagnostic, la localisation tumorale, la différentiation et le stade de la tumeur (Tableau 1). Nos résultats suggèrent que ce polymorphisme n´est pas un facteur prédictif de ces paramètres. Nos résultats étaient concordants avec ceux de la série de Carmen S.P. Lima *et al*. [16] et Toffoli *et al*. [17]. Cependant Park *et al*. [18] a mis en évidence une corrélation entre l´homozygote TT et la présence de métastases ganglionnaires avec une différence statistiquement significative (P = 0,003). Dans notre série, les sujets porteurs de l´allèle T (homozygote TT et hétérozygote CT) semblent être plus prédisposés à développer un CCR sporadique que les homozygotes CC (Tableau 2). Les résultats de notre série se rapprochent de celle de guimaraes JL *et al*. qui a montré un risque élevé de développer un CCR sporadique dans la population brésilienne pour la combinaison des génotypes CT et TT seule ou associée à d´autre polymorphisme impliqué dans le métabolisme des folates [19].

Le même résultat a été trouvé par l´équipe Prasad *et al*. où le génotype CT est associé à un risque accrue de développer un CRC (OR de 2.35, 95% CI 1.02-5.415) [20], ainsi que par l´équipe de El Awady *et al*. dans la population égyptienne avec un risque accrue de CCR pour les génotype CT et TT [21]. L´étude du risque relatif (RR) chez nos patients a trouvé un risque accru de CCR chez les sujets porteurs de l´allèle T ce qui suggère que les sujets ayant au moins un allèle 677T sont plus susceptible de développer un CCR sporadique dans notre population (Tableau 3). Une méta-analyse réalisée en 2013 sur 29783 cas et 41772 témoins par Mengmeng Zhao *et al*. a montré que l'allèle T peut avoir un rôle de protection contre le CCR [22]. Cette discordance pourraient s'expliquer en partie par la nécessité de prendre en compte les facteurs environnementaux tels que la région géographique, l'apport alimentaire en folate et le taux d'homocystéine. En effet, on sait que Le métabolisme des folates dépend non seulement des déterminants génétiques mais aussi de l´apport alimentaire en folate. Il est maintenant admis que l'insuffisance d'apport en folate augmente le risque de développer des lésions pré-néoplasique et donc le risque de CCR [23]. La consommation de légumes et de fibres alimentaire, et l´activité physique joue un rôle protecteur dans la survenue du CCR, par contre la prise d´alcool et le tabagisme ont un rôle délétère [8,24]. Enfin, l´étude du polymorphisme MTHFR est non seulement importante pour prédire le risque de survenue de CCR sporadique mais elle aurait un impact dans sa prise en charge thérapeutique en permettant de mieux comprendre la toxicité médicamenteuse liée à certaines chimiothérapies utilisées dans le CCR et d´expliquer une meilleure réponse de la tumeur au 5-fluorouracil [25].

## Conclusion

Notre étude a confirmé que Le polymorphisme C677T du gène MTHFR est un facteur de risque de susceptibilité génétique au CCR sporadique dans la population marocaine. il serait très intéressant d´étudier l´effet du polymorphisme de la MTHFR en prenant en considération les différents paramètres environnementaux pour mieux comprendre les mécanismes de la cancérogénèse colorectale liés aux folates et aux perturbations des enzymes qui les métabolisent.

### Etat des connaissances sur le sujet

- Le cancer colorectal représente un problème majeur de santé publique dans le monde;
- Rôle des folates dans la synthèse et la méthylation de l´ADN qui interfère avec les phénomènes de carcinogenèse;
- La méthylène tétrahydrofolate réductase est une enzyme clé dans le métabolisme des folates.

### Contribution de notre étude à la connaissance

- Le polymorphisme MTHFR C677T est un facteur de risque de cancer colorectal sporadique dans la population étudiée;
- L´homozygote TT n´est pas un facteur protecteur de CCR dans notre étude;
- Le polymorphisme MTHFR C677T n´est pas un facteur prédictif des caractéristiques clinico-pathologiques du CCR.

## References

[1] Global data health exchange (2016). Morocco-cancer registry of greater Casablanca region 2005 2006 2007. Global data health exchange.

[2] Giovannucci E, Willett WC (1994). Dietary factors and risk of colon cancer. Annals of medicine.

[3] Duthie SJ, Narayanan S, Blum S, Pirie L, Brand GM (2000). Folate deficiency in vitro induces uracil misincorporation and DNA hypomethylation and inhibits DNA excision repair in immortalized normal human colon epithelial cells. Nutrition and cancer.

[4] Leclerc D, Campeau E, Goyette P, Adjalla CE, Christensen B, Ross M (1996). Human methionine synthase: cDNA cloning and identification of mutations in patients of the cblG complementation group of folate/cobalamin disorders. Human molecular genetics.

[5] Lucock M (2000). Folic acid: nutritional biochemistry, molecular biology, and role in disease processes. Molecular genetics and metabolism.

[6] Stern LL, Mason JB, Selhub J, Choi SW (2000). Genomic DNA hypomethylation, a characteristic of most cancers, is present in peripheral leukocytes of individuals who are homozygous for the C677T polymorphism in the methylenetetrahydrofolate reductase gene. Cancer epidemiology, biomarkers & prevention: a publication of the American Association for Cancer Research, cosponsored by the American Society of Preventive Oncology.

[7] Giovannucci E, Rimm EB, Ascherio A, Stampfer MJ, Colditz GA, Willett WC (1995). Alcohol, low-methionine-low-folate diets, and risk of colon cancer in men. Journal of the National Cancer Institute.

[8] Su LJ, Arab L (2001). Nutritional status of folate and colon cancer risk: evidence from NHANES I epidemiologic follow-up study. Annals of epidemiology.

[9] Heijmans BT, Boer JM, Suchiman HE, Cornelisse CJ, Westendorp RG, Kromhout D (2003). A common variant of the methylenetetrahydrofolate reductase gene (1p36) is associated with an increased risk of cancer. Cancer research.

[10] Ma J, Stampfer MJ, Giovannucci E, Artigas C, Hunter DJ, Fuchs C (1997). Methylenetetrahydrofolate reductase polymorphism, dietary interactions, and risk of colorectal cancer. Cancer research.

[11] Pereira AC, Schettert IT, Morandini Filho AA, Guerra-Shinohara EM, Krieger JE (2004). Methylenetetrahydrofolate reductase (MTHFR) c677t gene variant modulates the homocysteine folate correlation in a mild folate-deficient population. Clinica chimica acta; international journal of clinical chemistry.

[12] Otani T, Iwasaki M, Hanaoka T, Kobayashi M, Ishihara J, Natsukawa S (2005). Folate, vitamin B6, vitamin B12, and vitamin B2 intake, genetic polymorphisms of related enzymes, and risk of colorectal cancer in a hospital-based case-control study in Japan. Nutrition and cancer.

[13] Chandy S, Sadananda Adiga MN, Ramachandra N, Krishnamoorthy S, Ramaswamy G, Savithri HS Association of methylenetetrahydrofolate reductase gene polymorphisms & colorectal cancer in India. The Indian journal of medical research. 2010 May;.

[14] Chen J, Giovannucci E, Kelsey K, Rimm EB, Stampfer MJ, Colditz GA (1996). A methylenetetrahydrofolate reductase polymorphism and the risk of colorectal cancer. Cancer research.

[15] Huang Y, Han S, Li Y, Mao Y, Xie Y (2007). Different roles of MTHFR C677T and A1298C polymorphisms in colorectal adenoma and colorectal cancer: a meta-analysis. Journal of human genetics.

[16] Lima CS, Nascimento H, Bonadia LC, Teori MT, Coy CS, Goes JR (2007). Polymorphisms in methylenetetrahydrofolate reductase gene (MTHFR) and the age of onset of sporadic colorectal adenocarcinoma. International journal of colorectal disease.

[17] Toffoli G, Gafa R, Russo A, Lanza G, Dolcetti R, Sartor F (2003). Methylenetetrahydrofolate reductase 677 C-->T polymorphism and risk of proximal colon cancer in north Italy. Clinical cancer research: an official journal of the American Association for Cancer Research.

[18] Park KS, Mok JW, Kim JC (1999). The 677C > T mutation in 5,10-methylenetetrahydrofolate reductase and colorectal cancer risk. Genetic testing.

[19] Guimaraes JL, Ayrizono Mde L, Coy CS, Lima CS (2011). Gene polymorphisms involved in folate and methionine metabolism and increased risk of sporadic colorectal adenocarcinoma. Tumour biology: the journal of the International Society for Oncodevelopmental Biology and Medicine.

[20] Prasad VV, Wilkhoo H (2011). Association of the functional polymorphism C677T in the methylenetetrahydrofolate reductase gene with colorectal, thyroid, breast, ovarian, and cervical cancers. Onkologie.

[21] El Awady MK, Karim AM, Hanna LS, El Husseiny LA, El Sahar M, Menem HA (2009). Methylenetetrahydrofolate reductase gene polymorphisms and the risk of colorectal carcinoma in a sample of Egyptian individuals. Cancer biomarkers: section a of disease markers.

[22] Zhao M, Li X, Xing C, Zhou B (2013). Association of methylenetetrahydrofolate reductase C677T and A1298C polymorphisms with colorectal cancer risk: a meta-analysis. Biomedical reports.

[23] Houlston RS, Tomlinson IP (2001). Polymorphisms and colorectal tumor risk. Gastroenterology.

[24] Sandhu MS, White IR, McPherson K (2001). Systematic review of the prospective cohort studies on meat consumption and colorectal cancer risk: a meta-analytical approach, Cancer epidemiology, biomarkers & prevention: a publication of the American Association for Cancer Research, cosponsored by the American Society of Preventive Oncology.

[25] Gusella M, Frigo AC, Bolzonella C, Marinelli R, Barile C, Bononi A (2009). Predictors of survival and toxicity in patients on adjuvant therapy with 5-fluorouracil for colorectal cancer. British journal of cancer.

